# Immunological Processes in the Orbit and Indications for Current and Potential Drug Targets

**DOI:** 10.3390/jcm13010072

**Published:** 2023-12-22

**Authors:** Katarzyna Cieplińska, Emilia Niedziela, Aldona Kowalska

**Affiliations:** 1 Department of Tumor Markers, Holy Cross Cancer Center, 25-734 Kielce, Poland; 2 Collegium Medicum, Jan Kochanowski University in Kielce, 25-317 Kielce, Poland; emilia.niedziela@onkol.kielce.pl (E.N.); aldona.kowalska@onkol.kielce.pl (A.K.); 3 Department of Endocrinology, Holy Cross Cancer Center, 25-734 Kielce, Poland

**Keywords:** thyroid eye disease (TED), drug targets, immunological process

## Abstract

Thyroid eye disease (TED) is an extrathyroidal manifestation of Graves’ disease (GD). Similar to GD, TED is caused by an autoimmune response. TED is an autoimmune inflammatory disorder of the orbit and periorbital tissues, characterized by upper eyelid retraction, swelling, redness, conjunctivitis, and bulging eyes. The pathophysiology of TED is complex, with the infiltration of activated T lymphocytes and activation of orbital fibroblasts (OFs) and autoantibodies against the common autoantigen of thyroid and orbital tissues. Better understanding of the multifactorial pathogenesis of TED contributes to the development of more effective therapies. In this review, we present current and potential drug targets. The ideal treatment should slow progression of the disease with as little interference with patient immunity as possible. In the future, TED treatment will target the immune mechanism involved in the disease and will be based on a strategy of restoring tolerance to autoantigens.

## 1. Introduction

Graves’ disease (GD) is an organ-specific autoimmune disease characterized by loss of T lymphocyte tolerance to self-antigens, most often to the thyroid-stimulating hormone receptor (TSHR). Activated CD4^+^ helper T cells and subsequent production of cytokines stimulates B cells to produce autoantibodies that bind to the TSHR (i.e., anti-TSHR-Ab); binding of these antibodies mimics the action of thyrotropin (TSH), leading to stimulation of thyroid hormone secretion [1,2,3,4].

**Figure 1:**
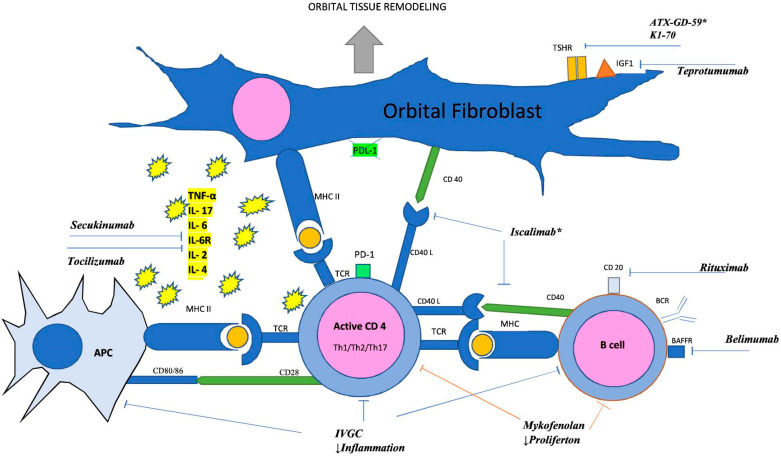
Immunological Processes in the Orbit and Indications for Current and Potential Drug Targets. *, drugs potentially useful in TED so far without studies to confirm their effectiveness; APC, antigen-presenting cell; BAFF, B-cell activating factor; BCR, B cell receptor; IGF-1R, insulin-like growth factor 1 receptor; IL-17, interleukin 17; IL-6, interleukin 6; IL-6R, interleukin 6 receptor; IL-2, interleukin 2; IL-4, interleukin 4; IVGC, intravenous glucocorticoid; MHC II, major histocompatibility complex class II; PD-1, programmed cell death protein 1; PD-L1, programmed death-ligand 1; TCR, T cell receptor; TNF-α, tumor necrosis factor α; TSHR, thyrotropin receptor.

Thyroid eye disease (TED) is an extrathyroidal manifestation of GD [5,6,7,8]. Studies show that TED occurs in 13–69% of GD cases [9,10]. Similar to GD, TED is caused by an autoimmune response. The pathophysiology underlying TED is complex, with key factors being the infiltration of activated T and B lymphocytes, activation of orbital fibroblasts (OFs), and the presence of autoantibodies targeting a common autoantigen (i.e., TSHR) expressed in thyroid and orbital tissues [6,7,9,10,11,12,13,14]. In TED, TSHR receptor antibodies (TRABs) and antibodies to insulin-like growth factor 1 receptor (anti-IGF-1R) interact with OFs. Cigarette smoking, thyroid dysfunction, a history of radioactive iodine therapy, and elevated levels of TRABs are now known to be the major modifiable risk factors for TED. Treatment of hypercholesterolemia in patients with GD is receiving growing interest, as it is also considered a risk factor for TED [15]. A better understanding of the intricate pathogenesis of TED may contribute to the development of new, safe, and effective treatment strategies (Figure 1).

## 2. Pathogenesis

### 2.1. T Lymphocytes

Studies of the pathogenesis of TED reveal involvement of T lymphocytes and their associated cytokines in the development and perpetuation of orbital inflammation [13,16,17]. T lymphocytes develop from lymphoid stem cells in the bone marrow and then mature in the thymus [18], where they undergo positive and negative selection followed by differentiation into CD4^+^ T lymphocytes or CD8^+^ T lymphocytes [13]. CD4^+^ T lymphocytes are helper T (Th) cells that play a leading role in cellular and humoral immunity [19]. The main role of cytotoxic CD8^+^ T lymphocytes is to destroy tumor cells and cells infected with intracellular pathogens [20,21]. Immunohistochemical analyses by Yuan-Ping Hai et al. showed that during the active phase of disease, orbital tissues are infiltrated mainly by activated CD4^+^ T cells, CD68^+^ macrophages, and, to a lesser extent, plasma cells and mast cells [22]. An Italian study reported a positive correlation between orbital T cell infiltration and clinical activity scores (CASs) [23].

Autotolerance, defined as an absence of immunological reactivity to self-antigens, is maintained by mechanisms that include clonal deletion, clonal anergy, active suppression, and antigen sequestration. Clonal deletion occurs in the thymus, where CD4^+^CD8^+^ double-positive cells recognize antigens presented in the context of the major histocompatibility complex (MHC). This process results in the positive selection of cells with a low affinity for MHC molecules. Unselected cells die by apoptosis, a process called “death by neglect”. Positively selected T cells interact with autopeptides, and if cells react to an antigen with high affinity, they undergo negative selection. Disruption of clonal deletion can lead to the release of autoreactive T cells, which can trigger autoimmune disease [24,25,26].

In TED, autotolerance is impaired. Antigen-presenting cells (APCs) recognize the autoantigen TSHR expressed on OFs and activate T lymphocytes [13,24]. The first signal for activation comes from the TCR receptor, through which the T cell recognizes a peptide presented on the surface of the APC (this peptide is derived from a previously digested protein and is presented on MHC molecules). Recognition of the antigenic determinant activates secretion of IL-2, followed by expression of the receptor for IL-2 (CD25) on the surface of the lymphocyte. The second signal for T lymphocyte activation originates from co-stimulatory molecules, including the binding of CD80/86 to CD28 on the T cell surface [16,25]. Cytokine signaling (IL-2/IL-2R) is the third component required for lymphocyte activation [25]. Activated T cells differentiate into subpopulations depending on the specific pathways and cytokines they encounter, which consist of the following: Th1 (supporting the cellular response), Th2 (supporting the humoral response), Th17 (involved in inflammatory diseases), and regulatory T (Treg) cells (demonstrating regulatory functions) [27,28,29,30].

The absence of interaction between co-stimulatory proteins on T cells and APCs, which normally occurs during autoantigen presentation, is related to the lack of a pathogenic signal from toll-like receptors, leading to reversible T cell anergy, which is another mechanism of autotolerance. The T cell response is regulated by a balance between co-stimulatory and co-inhibitory signals. Immune checkpoints are crucial for effective T cell activation, as well as for maintenance of self-tolerance, prevention from self-tissue destruction by the host immune system, and provision of protective immunity [31,32,33,34,35]. 

Cytotoxic T-lymphocyte associated protein (CTLA-4, CD152), which is expressed constitutively on Treg cells that play a key role in maintaining autotolerance through active suppression of immune responses, is an immune checkpoint responsible for negative regulation of T cell activation [36,37]. CTLA-4/CD152 has a much higher affinity for the B7 receptor (CD80/86) than CD28; therefore, it wins the competition and deprives lymphocytes of their main co-stimulatory factor. In this way, CTLA-4 transmits a T cell inhibitory signal.

As reported previously, expression of CTLA-4 mRNA in the orbital tissue of patients with severe TED is lower than in that of those with mild TED, supporting the involvement of CTLA-4 in TED autoimmunity [38]. 

Recent studies have reported a relationship between TED and the programmed cell death protein 1/programmed death-ligand 1 (PD-1/PD-L1) pathway. The PD-1 receptor, located on the surface of T cells, is another immune checkpoint that influences T cell exhaustion. PD-L1 is expressed mainly on various tumor cells [39,40]. PD-1/PD-L1 is the main negative co-stimulatory pathway that drives the immune tolerance of cancer cells. Zhibin Liu et al. reported the absence of PD-L1 in OFs, which might have been the reason for observed reduced orbital immune tolerance. In that study, the authors performed an experiment in which they cultured OFs together with T cells and then added exogenous PD-L1. Exogenous PD-L1 inhibited T cell activity, resulting in decreased activation of OFs and reduced expression of sICAM-1, IL-6, IL-8, and hyaluronan [41]. A 2023 Swedish study showed that soluble programmed death-ligand 1 (sPD-L1) was significantly elevated in TED patients compared to the group of patients with GD without TED [42]. These results confirm that PD-L1 may be involved in the pathogenesis of TED.

An increasing number of immune checkpoints have been identified. Blocking a co-inhibitor immune checkpoint molecule can trigger anti-cancer immunity, while stimulating the same molecule can reduce overreaction in autoimmune disease. Therefore, the question is whether such interventions would also be effective for TED without increasing the incidence of infections and/or cancers.

### 2.2. Cytokines and Chemokines

Previous studies have shown that the initial TED phase is characterized by increased activity of Th1 lymphocytes, which facilitate cellular immunity and produce mainly IFN-γ, IL-1β, IL-2, and TNF-α. The later inactive phase involves Th2 cells that release cytokines such as IL-4, IL-5, IL-10, and IL-13 [6,43,44] and is characterized by tissue remodeling and fibrosis [45].

IL-17A

More recent publications have reported that an increased number of Th17 cells, a subpopulation of CD4 helper lymphocytes, was found in patients with TED [18,46,47]. Moreover, levels of IL-17A in the peripheral blood of TED patients were higher than those in controls [48,49,50]. In addition, a high level of IL-17A was detected in the tears of TED patients [51,52]. Immunohistochemical evaluation of orbital tissues revealed increased expression not only of IL-17A, but also of cytokines (TGF-β, IL-6, IL-1β, and IL-23A) that promote the differentiation of naive CD4 lymphocytes into Th17 cells [50]. IL-17A promotes inflammation and fibrosis in TED patients by acting on OFs, and alongside CD40L, it enhances the expression and secretion of RANTES by OFs [53]. Fang et al. found a possible relationship between IL-17 and OFs. IL-17 stimulated the expression of pro-inflammatory cytokines (IL-6, IL-8, MCP-1, TNF-α, and GM-CSF) and co-stimulatory molecules (CD40 and MHC II) on OFs, thereby affecting fibrosis and adipogenesis [47,48,49,50]. In addition, OFs influenced the differentiation and function of Th17 cells by secreting prostaglandin E2 (PGE2) [47]. Therefore, IL-17A may therefore be a therapeutic target in TED.

RANTES and IL-16

Regulated on activation, normal T cell expressed and secreted (RANTES) and IL-16 are chemokines that impact cell migration [24,25]. Studies show that RANTES and IL-16 are elevated in the serum and orbital tissue of TED patients [54]. Thus, IL-16 and RANTES may be involved in orbital tissue inflammation in TED by attracting lymphocytes [3,7,13].

### 2.3. Fibroblasts

In TED, OFs are the cells targeted by the immune response [6,7,9,12,13,55,56,57]. The interaction between OFs and immune cells, mediated by various cytokines, chemokines, and lipid mediators, is the fundamental mechanism that sustains orbital inflammation [7,11,56,58]. Based on the expression of the CD90 molecule, OFs have been classified as CD90^+^ or CD90^−^ subtypes [59]. CD90^−^ OFs differentiate into adipocytes in response to IL-1, IL-6 [60], and prostaglandin D2 [61], and they induce adipogenesis [62]. CD90^+^ OFs differentiate into myofibroblasts in the presence of transforming growth factor beta (TGF-β), and they subsequently cause orbital tissue remodeling and fibrosis [3,56,59]. Proliferation of fibroblasts plays a crucial role in tissue remodeling and fibrosis [60,63]. In TED, autoreactive T cells boost proliferation of OFs [64], as well as production of glycosaminoglycans, resulting in edema and increased orbital tissue volume [54,65]. OFs produce IL-6, IL-8, IL-16, and monocyte chemoattractant protein-1 (MCP-1), all of which play a role in orbital inflammation [66]. Increased levels of cytokines stimulate OFs to produce intercellular adhesion molecules, which are also involved in local inflammation [3]. OFs may also function as facultative APCs and stimulate autoreactive T cells. This thesis is supported by the fact that orbital connective tissues in TED express higher gene and protein levels of MHC class II and CD40 molecules than control tissues [16]. In addition, OFs activate T cells through CD40–CD40L signaling, thereby increasing orbital inflammation [16,56,64].

### 2.4. TSHR

TSHR-stimulating antibodies and T cell infiltration have been identified as causative factors of autoimmune thyroid disease (i.e., GD) [3,4]. Since mRNA encoding TSHR was detected in the orbital tissue of TED patients [67], and TED symptoms occur within 18 months of a diagnosis of GD, a correlation between these two conditions was concluded. Both diseases may share a common mechanism, and the TSHR autoantigen plays a role [9,68]. Some studies report a correlation between ocular changes and the level of TSHR-stimulating antibodies (i.e., thyroid-stimulating immunoglobulins, TSIs) [14,15]. 

### 2.5. Insulin-like Growth Factor 1 Receptor (IGF-1R)

IGF-1R is another autoantigen involved in the pathogenesis of TED [11,69]. Recent studies have reported that expression of the IGF-1R protein is higher in orbital tissue from TED patients than in that from controls [70,71]. However, despite the fact that many previous studies confirmed the involvement of IGF-1R in TED, activation of IGF-1R on OFs after binding by stimulating antibodies (IGF-1R-Ab) remains controversial [56]. It is now believed that TSHR and IGF-1R form a functional complex on the surface of OFs and that activation of the IGF-1R pathway is triggered by TSHR stimulation rather than by direct interaction with IGF-1R [72].

### 2.6. B Lymphocytes

B lymphocytes are precursors of plasma cells. The cells function as specialized APCs, although they require a signal from an activated CD4^+^ Th2 cell to respond to a T-dependent antigen. An antigen binds to the B cell receptor (BCR; from which the first activation signal originates) on the surface of a B lymphocyte. Then, the antigen is pinocytosed into the cell, where it is processed and presented in the context of MHC II molecules. The CD4^+^ lymphocyte recognizes the presented peptide via its TCR and, once the subsequent activation conditions are met, transmits the second signal, which is generated via combination of CD40L (CD154) on the activated CD4^+^ Th2 lymphocyte and the CD40 molecule on the B lymphocyte (i.e., the APC) [13,25,26]. Then, the activated T cells secrete IL-4, which is important for further activation of CD19^+^ B cells and immunoglobulin class switching [7,23]. Rituximab, which is a useful treatment for TED, targets the CD20 antigen of the B lymphocyte.

### 2.7. CD40/CD154

Earlier studies have shown that CD40 is also expressed on OFs in TED and that CD40 levels are higher in TED samples than in controls [66]. More recent studies have reported higher expression of CD40 on thyrocytes from patients with hyperthyroidism than by control cells [73].

Taking into consideration the proven role of the CD40–CD40L pathway in activating CD4^+^ lymphocytes, antibody production by plasma cells, activation of APCs, secretion of cytokines, and increased expression of co-stimulatory molecules [74], this pathway should be considered as a target for TED therapy.

### 2.8. BAFF

B-cell activating factor (BAFF) is a B cell survival factor that promotes the maturation and production of immunoglobulins [75,76,77]. BAFF supports autoreactive B lymphocytes and prevents their deletion. High serum levels of BAFF were reported in patients with TED [72]. Expression of the BAFF protein in the orbital tissue of TED patients is higher than that in healthy controls [71].

## 3. Potential and Current Drug Targets

Currently available treatments are not sufficient in terms of efficacy and safety, especially in the context of moderate-to-severe TED. Individuals with mild TED do not require systemic therapies. The primary aim of new treatments for TED is to target the main autoantigens that trigger the disease, as well as the molecules that play a key role in its pathogenesis (Table 1).

### 3.1. Glucocorticoids (GCs) 

Intravenous GCs (IVGCs) are the most popular drugs used to treat TED; this is because they are easily available and because access to newly developed biological drugs (teprotumumab, which is registered only in the USA) is limited.

IVGCs are the first-line treatment for TED in Europe. Steroids reduce orbital inflammation during the active phase of the disease, but they have little effect on diplopia and exophthalmos [23,78,79,80].

GCs inhibit the activity of phospholipase A2, as well as expression of various cytokines and pro-inflammatory proteins. They also reduce the number of circulating T and B lymphocytes, and the number of basophils and eosinophils. GCs also regulate the activity and number of APCs (dendritic cells). They act through a genomic mechanism (cytoplasmic and nuclear) to regulate the expression of genes and through a non-genomic mechanism via cell membrane receptors, thereby inducing rapid changes in the activity of many signaling pathways [78,81].

Because TED tends to improve spontaneously, a lack of placebo-controlled trials is a significant issue. IVGCs are associated with significant side effects. Indeed, steroid therapy is associated with carbohydrate metabolism disorders, hypertension, bone loss, Cushing’s syndrome, psychiatric events, exacerbation of tuberculosis and hepatitis B, and systemic immunodeficiency. Patients treated with oral GCs had the highest rate of adverse events, while IVGC was associated with rare, but more severe cases like hepatotoxicity, most of which were dose-dependent (>8–10 g per cycle). Individual cases of cardiovascular complications (pulmonary edema, pulmonary embolism, myocardial infarction and coronary thrombosis, cerebro–cerebral artery obstruction) have also been reported [82]. To avoid severe adverse events, the European Graves’ Orbitopathy Group (EUGOGO) recommends a maximum cumulative dose of 8 g of i.v. methylprednisolone per treatment cycle and a maximum of 750–1000 mg per day. However, the usual doses used are about 3.5–4.5 g of methylprednisolone administered intravenously in 12 weekly infusions. Oral GCs are usually initiated in monotherapy at a dose of 50–100 mg/day, gradually tapered to low doses over up to 6 months, or used as a combination therapy of parenteral and oral GCs, when the recommended scheme is inconvenient for the patient [5,82,83]. 

A 2022 metanalysis showed that IVGC treatment (4.5–5 g over 12 weeks) resulted in a proptosis reduction of only 0.16 mm from baseline compared with the placebo. Meanwhile, the teprotumumab therapy resulted in unprecedented reduction of the exophthalmos (−2.31 mm from baseline). Randomized trials comparing the two treatments are needed to establish the optimal therapeutic approach [84]. The recent consensus statement jointly issued by the American Thyroid Association (ATA) and the European Thyroid Association (ETA) recommended IVGC therapy as a preferred treatment for active moderate-to-severe TED when disease activity is the dominant feature in the absence of significant proptosis or diplopia [85].

### 3.2. Mycophenolate Mofetil (MMF)

MMF, a prodrug of mycophenolic acid, inhibits inosine monophosphate dehydrogenase 2 (IMPDH2) in activated B and T lymphocytes, thereby reducing lymphocyte proliferation and antibody production. IMPDH2 is a critical enzyme required for the formation of purine nucleotides in T and B lymphocytes (other tissues can use alternative pathways) [86,87]. A multicenter study conducted by the EUGOGO compared the efficiency and safety of MMF plus IVGC therapy with that of standard IVGC monotherapy. A post hoc analysis at week 24 revealed a better response to therapy in the combination group (71%) than in the IVGC-only group (53%). In the combination group, adverse effects were mild, and there was no increased risk of infection. However, the short follow-up time and limited number of participants are limitations of this trial [88]. An analysis of MMF safety data from this study showed that MMF is well tolerated as both a monotherapy and as combination therapy. A study analyzing the efficacy of oral MMF with GC administration in the experimental group vs. GC alone in the control group, in patients with moderate-to-severe TED, showed better improvement in clinical outcomes (reduction in the CAS score by ≥2 points; reduction in the lid width by ≥2 mm; reduction in proptosis by ≥2 mm; improvement by ≥1 grade in soft tissue involvement, such as eyelid or conjunctival redness and eyelid or conjunctival edema; reduction in the diplopia grade by ≥1 grade) at 12 and 24 weeks in the experimental group (73.3% and 83.3%) than in the control group (46.7% and 60.0%) (*p* < 0.05). In addition, levels of immune markers such as TRAB, IL-6, and the CD4^+^/CD8^+^ ratio in the experimental group were significantly lower than those before treatment and in the control group after 24 weeks of treatment. This suggests a greater efficiency of the combined therapy than IVGCs alone [89]. 

The results of the above studies resulted in a recommendation by the EUGOGO published in 2021 which introduced high-dose GCs in combination with mycophenolate (MMF) as a first-line treatment [5].

### 3.3. Therapies Targeting Immune Checkpoints (ICPs)

ICPs maintain immune tolerance and suppress autoimmunity. The role of ICPs was demonstrated in animal models and confirmed by the clinical success of ICP-targeted drugs for autoimmune diseases [36,90]. Recent clinical observations suggest that ICP blockade can cause autoimmune disorders, termed immune-related adverse events (irAEs). IrAEs were reported in up to 80% of patients receiving these therapies. Indeed, increased T lymphocyte activity, as well as elevated levels of inflammatory cytokines and pre-existing autoantibodies, are the probable causes of this phenomenon [91]. A syndrome of orbital inflammation similar to TED was reported in patients without a history of thyroid disease who were treated with anti-CTLA-4 or anti-PD-1 [92,93,94,95]. Although most patients had normal thyroid function with elevated anti-thyroid antibodies (especially anti-thyroid peroxidase and anti-thyroglobulin, sometimes anti-TSHR), some developed hyperthyroidism [92]. Most patients were treated successfully with systemic steroids. Recurrence and persistence of eye symptoms after discontinuation of steroids were also encountered [94].

Considering the key role of ICPs in immune tolerance, they have a potential utility as therapeutic targets to rescue immune tolerance and treat autoimmune diseases by blocking activating receptors or by stimulating inhibitory receptors [36,90]. However, such non-selective interference with the immune system may cause side effects such as a reduction in resistance to pathogens, as well as an increased incidence of cancer. More research is needed to examine the effectiveness and safety of such drugs. It should be noted that there are currently no published studies on the use of targeted ICP therapies in TED.

The most effective drug is abatacept, which is based on the soluble ICP receptor. Abatacept is a fusion protein comprising the extracellular domain of human CTLA-4 and the fragment crystallizable (Fc) domain of human IgG1. Abatacept is approved for the treatment of adult patients with highly active, progressive rheumatoid arthritis (RA) not previously treated with methotrexate, and for the treatment of active rheumatoid arthritis (moderate-to-severe) in adult patients who show an inadequate response to previous treatment or intolerance to current treatment with one or more disease-modifying antirheumatic drugs or inhibitors of tumor necrosis factor (anti-TNF) [96].

### 3.4. Rituximab (RTX)

RTX is a B cell-depleting IgG1 monoclonal immunoglobulin against the CD20 antigen expressed by B lymphocytes. Binding to CD20 activates its complement, resulting in the lysis of plasma cell precursors and a subsequent reduction in autoantibody levels. Rituximab also inhibits antigen presentation, cytokine release, and co-stimulatory signaling between B and T lymphocytes [97].

Previous studies have reported conflicting results regarding the efficacy of rituximab as a treatment for TED. An Italian study found that RTX (1 g, twice, at a 2-week interval or a single dose of 500 mg) was more effective at reducing CAS than IVGCs administered once a week (7.5 g cumulative dose) [97]. By contrast, an American study of 25 patients did not provide any evidence that RTX (2 g) therapy is more effective than the placebo [98]. A study from France also failed to confirm the efficacy of RTX as an effective treatment for moderate-to-severe steroid-resistant TED with a long disease duration [99]. RTX was associated with the risk of a severe reaction to the infusion, manifested by a rapid onset of orbital edema and subsequent visual deterioration, controlled after the administration of 100 mg of intravenous hydrocortisone and resolving spontaneously. Most patients treated with RTX experienced mild reactions to the infusion, characterized by an itchy throat and nasal congestion. These symptoms occurred during first RTX administration and resolved spontaneously in all patients after slowing the RTX infusion or administering 100 mg of intravenous hydrocortisone. One patient had elevated blood pressure values, which normalized over time [97]. In a study by Stan et al., RTX side effects included skin symptoms (itching, rash), vasculitis, optic neuropathy, severe lacrimation, and gastrointestinal complaints [98]. 

In 2022, the ATA and the ETA jointly stated that RTX may be considered for TED inactivation in GC-resistant patients with active, moderate-to-severe TED, particularly with evident soft tissue involvement [85].

### 3.5. Belimumab

A recently published study (EudraCT 2015-002127-26) compared IVGCs with belimumab (BMB), a fully human IgG1 monoclonal antibody against BAFF (B-cell activating factor) that has been approved for the treatment of systemic lupus erythematosus (SLE)*,* as a treatment for active moderate-to-severe TED. Over the course of the study, 14 patients received intravenous BMB (on days 0, 14, and 28 and then every 4 weeks thereafter for five infusion cycles) and another 13 patients received i.v. methylprednisolone (ivMP) (833 mg weekly for six cycles, followed by a single cycle of 425 mg/week). CAS decreased significantly in both groups after 24 weeks; however, the study results indicated that the decrease related to BMB was slightly slower than that related to ivMP. After 12 weeks, patients receiving IVGCs had significantly lower CAS than patients receiving BMB. The study reported a good tolerability of the BMB treatment. Thus, BMB is an excellent alternative to IVGCs when the latter is contraindicated or ineffective [100].

The treatment of seropositive SLE with belimumab has generally been well tolerated. However, reported side effects include infections, infusion reactions, hypersensitivity, headaches, nausea, and fatigue. Rarer side effects include psychiatric illnesses, neutropenia, thrombocytopenia, and hypogammaglobulinemia. Progressive multifocal leukoencephalopathy has been reported with BMB treatment. BMB may increase the risk of malignancy. Caution should be exercised when considering BMB therapy for patients with a history of malignancy or when considering continuing treatment in patients who develop malignancy [101,102].

### 3.6. Iscalimab

Iscalimab, a fully human anti-CD40 monoclonal antibody that disrupts CD40–CD154 interactions, has shown promising results in patients with Graves’ hyperthyroidism. Common adverse events include fatigue, nausea, headache, insomnia, upper respiratory tract infection, and cystitis [103].

The fact that CD40 receptors are also present on OFs implies a beneficial effect on TED [66]. So far, drugs targeting CD40 have not been studied for the treatment of TED.

### 3.7. Secukinumab

Secukinumab is a recombinant, fully human, monoclonal anti-IL-17A antibody approved for the treatment of three inflammatory/autoimmune diseases: moderate-to-severe plaque psoriasis (PsO), psoriatic arthritis (PsA), and axial spondylarthritis (axSpA). A multicenter phase 3 study investigated the efficacy and safety of secukinumab at a dose of 300 mg in adult patients with active, moderate-to-severe TED, with a placebo-controlled 16-week treatment period and a follow-up period (NCT04737330). In July 2023, the study was terminated before completion of the study. Unfortunately, analysis of blinded patient data showed a very low probability of the study meeting the primary efficacy endpoints. No safety concerns were identified. Adverse effects occurring in patients treated with secukinumab for psoriasis include the following: *Candida* infections, erosio interdigitalis blastomycetica, fatigue, nasopharyngitis, paradoxical arthritis, bronchitis, arthralgia, pruritus, and weight gain [104].

### 3.8. Tocilizumab 

Tocilizumab (TCZ) is a humanized monoclonal anti-IL-6R antibody. IL-6 is a pro-inflammatory cytokine that induces the development of Th17 lymphocytes and inhibits differentiation of Treg [49,105]. IL-6 stimulates TSHR expression by OFs from TED patients [106]. Higher concentrations of IL-6 were present in the tears of TED patients than in those of the controls. Moreover, there is a positive correlation between IL-6 concentration and CAS [107]. In addition, GD patients with symptoms of TED had higher serum IL-6 concentrations than patients without TED symptoms [108].

A study investigating the efficacy of TCZ in patients with moderate-to-severe corticosteroid-resistant TED was conducted in Spain [109]. The double-blind, randomized clinical trial enrolled 32 adults with TED from 10 medical centers. Patients were administered intravenous TCZ (8 mg/kg body weight) or a placebo at weeks 0, 4, 8, and 12. Follow-up was conducted for an additional 28 weeks. The primary outcome was the percentage of patients in whom CAS changed from baseline by at least 2 points by week 16.

The primary endpoint was achieved in 93.3% of patients receiving TCZ and in 58.8% of patients receiving the placebo. Moreover, there was also a significant difference in the percentage of patients who achieved a CAS < 3 (86.7% for tocilizumab vs. 35.2% for placebo) at week 16. In addition, more patients in the TCZ-treated group had an improved EUGOGO-proposed composite ophthalmic score (73.3% vs. 29.4% in the placebo group) at 16 weeks and an improvement in their exophthalmos from baseline to week 16 (−1.5 mm vs. 0.0 mm, respectively). During the study, two patients experienced serious side effects caused by the TCZ therapy. One had a moderate increase in transaminases at week 8, whereas the other had acute pyelonephritis at week 32.

Further observational studies showed the efficiency of TCZ therapy in patients with GC-refractory TED, demonstrating a decrease in CAS and TSI levels and acceptable treatment tolerance [110,111]. The results of a 2023 Greek study showed that TCZ significantly reduced the median CAS by 3 units (*p* = 0.002), TSI level by 11.02 IU/l (*p* = 0.006), proptosis of the right eye by 2.3 mm (*p* = 0.003), and proptosis of the left eye by 1.6 mm (*p* = 0.002), measured by a Hertel exophthalmometry. Diplopia persisted in fewer patients (25%, *p* = 0.250) after TCZ treatment [112]. A meta-analysis published in 2023, which analyzed 12 trials with 488 patients, confirmed the high treatment response rate of TCZ, including proptosis reduction (with a better outcome than TPT) [113]. An ongoing multicenter trial aims to evaluate the efficacy of TED treatment with intravenous TCZ compared to IVGCs (EudraCT number: 2018-002790-22, ClinicalTrials.gov (accessed on 1 November 2023) identifier: NCT04876534).

### 3.9. K1-70 Targeting of the Pathophysiology of Major GD and TED Antigens

Due to the presence of TSHR on OFs [69] and its identification as an autoantigen in GD and TED, it seems that blocking the TSHR is a promising targeted therapy. The TSHR antagonist K1-70 blocks the binding site for thyroid-stimulating immunoglobulin (TSI). Clinical study NCT02904330, which evaluated the safety and tolerability of K1-70 in GD patients [114], found an improvement in thyroid function parameters and TED symptoms (i.e., reduced exophthalmos, reduced photosensitivity). The commonly reported adverse events (particularly with higher doses of K1-70) were fatigue and lethargy. No deaths or other serious adverse events that were significant were reported. Hypothyroidism was the expected effect of K1-70 and was managed by standard care. It was not considered an adverse event [114]. 

It should be emphasized that these are preliminary results of a phase I study and as such need to be consolidated in further clinical trials.

### 3.10. Teprotumumab (TPT)

TPT is a fully human monoclonal antibody that binds to and blocks the extracellular part of IGF-1R. This leads to the internalization and degradation of IGF-1R, thereby reducing expression of the receptor on the cell surface [115]. TPT is the first pharmacological treatment for TED that shows comprehensive efficacy (in terms of disease activity, severity, and quality of life) as well as a favorable safety profile. Thus, in January 2020, TPT was the first drug approved by the U.S. Food and Drug Administration for the treatment of TED in adults. The safety and efficacy of TPT were evaluated in two randomized, placebo-controlled, multicenter studies. The phase 2 study included 88 patients, and the phase 3 study enrolled 83 patients, with active moderate-to-severe TED [116,117]. The studies had a similar design, with patients randomized to a TPT-treated group (*n* = 83) and a placebo group (*n* = 87). Taking both phases into account, 73% of patients in the TPT groups (vs. 14% in the placebo groups) responded, with improvements both in CAS and exophthalmos.

Improvements in proptosis of ≥2 mm occurred in 77% of patients in the TPT-treated groups and in 15% of patients in the placebo group at week 24. TPT was well tolerated, and adverse events were mostly of mild-to-moderate severity. The only adverse event in the phase 2 study unequivocally associated with TPT was hyperglycemia, which occurred in 7/84 patients treated with TPT (8.3%). This mainly affected patients with pre-existing diabetes and was controlled after modification of their diabetes treatment. In the phase 3 study, most adverse reactions were mild or moderate. During the trial, two serious adverse events were reported in the TPT group, one of which (an infusion reaction) led to treatment discontinuation.

Common side effects of TPT include nausea, diarrhea, muscle spasms, dysgeusia, headaches, dry skin, infusion reactions, alopecia, paresthesia, weight loss, and hyperglycemia [118].

After FDA drug approval, one case of amyloid encephalopathy and several cases of onset or exacerbation of inflammatory bowel disease were reported [118,119,120]. Sensorineural hearing impairment has been reported in 15% of patients treated with teprotumumab, being permanent in 45% of them, with no clear link to pre-existing otological problems [121].

In the 2022 OPTIC-X trial, patients not responding to TPT in the prior OPTIC trial or those with disease exacerbation were treated for the first time (as previous placebo patients) or retreated with TPT. Most patients achieved a response to therapy. Thirty-three out of 37 OPTIC placebo patients achieved an exophthalmos reduction after TPT treatment in the OPTIC-X study [122]. In addition, this study showed that TPT was effective in patients who had had the disease for more than a year. Patients with chronic, inactive disease were also reported to benefit from TPT [123,124].

The ETA and ATA recommend the treatment of moderate-to-severe TED with TPT if the drug is available. TPT should be the first choice for patients whose main symptom is proptosis and/or diplopia [85]. In April 2023, the FDA approved an update to the indications for teprotumumab-trbw (TEPEZZA^®^, Horizon Therapeutics, Dublin, Ireland), specifying its use in all patients with TED, regardless of TED activity or duration. Horizon Therapeutics presented its data from a phase IV clinical study (NCT04583735) investigating the efficacy and safety of teprotumumab-trbw in the treatment of patients with chronic inactive TED (in adults who had lived with TED for 2 to 10 years prior to the study and had low CAS) at the 2023 Annual Meeting of the Endocrine Society in Chicago. The data showed that TPT treatment was associated with a reduction in proptosis of 2.41 mm from baseline compared with 0.92 mm in those receiving a placebo (*p* = 0.0004). In addition, 62% of teprotumumab-treated patients experienced clinically significant improvement at week 24 (≥2 mm) compared with 25% of placebo-treated patients (*p* = 0.0134).

### 3.11. ATX-GD-59 Restoration of Tolerance

Restoration of immune tolerance to TSHR, IGF-1R, and potentially other relevant autoantigens involved in the pathogenesis of GD and TED remains the main goal for many researchers due to the lack of generalized immunosuppression. Specific immunotherapy, known as allergology, requires the administration of an allergen (or antigen) in gradually increasing quantities until the desired level of immunological tolerance is achieved [125,126]. This type of therapy may also be effective in modifying the course of autoimmune disease by restoring immune tolerance to autoantigens. ATX-GD-59 (a combination of two TSHR peptides) was evaluated in a phase 1 study of 12 patients with previously untreated mild-to-moderate Graves’ hyperthyroidism, receiving 10 doses of ATX-GD-59 intradermally over an 18-week period [127]. Their concentrations of serum TSHR autoantibodies decreased during the study, which corresponded to changes in free thyroid hormone levels (r = 0.85, *p* = 0.002 for TSHR autoantibodies vs. free triiodothyronine). The most common side effects were mild swelling and pain at the injection site. The above study confirms the potential of ATX-GD-59 in Graves’ hyperthyroidism. To date, there are no studies on the efficacy of this agent in the treatment of TED. 

**Table 1:** Overview of different classes of drugs and their targets in thyroid eye disease (TED) used in current approved treatments, in ongoing clinical trials, and under investigation.

Drug	Mechanism of Action	Study Population	Design	Intervention	Main Findings
Mycophenolate mofetil (MMF) [88]	Inhibition of IMPDH2	164 patients with active moderate-to-severe Graves’ orbitopathy	Multicenter RCT	MMF with ivMP vs. ivMP alone	No significant difference in the rate of response at 12 weeks or rate of relapse at 24 and 36 weeks. The combination of MMF with ivMP improved the response rate by 24 weeks in patients with active and moderate-to-severe Graves’ orbitopathy.
Rituximab (RTX) [97]	Anti-CD20 MAb	32 patients with active moderate-to-severe TED	Single-center RCT	RTX vs. ivMP	RTX was more effective after 16, 20, and 24 weeks. Patients receiving RTX achieved improved motility after 52 weeks. There was no disease reactivation in the RTX group.
Rituximab (RTX) [98]	Anti-CD20 MAb	25 patients with active moderate-to-severe TED	Single-center RCT	RTX vs. placebo	There were no differences between the RTX and placebo groups in the proportion of patients showing CAS improvement after 24 weeks or in the decrease in CAS from baseline to 24 or 52 weeks.
Belimumab (BMB) [100]	Anti-BAFF MAb	27 patients with active, moderate-to-severe TED	Single-center RCT	BMB vs. ivMP	After 12 weeks, patients treated with ivMP had significantly lower CAS than patients treated with BMB. CAS decreased significantly in both groups after 24 weeks. Proptosis improved in all patients (*p* < 0.05), but improvement was greater in ivMP group than in patients treated with BMB (*p* < 0.04)
Secukinumab [NCT04737330]	Anti-IL-17A MAb	28 patients with active, moderate-to-severe TED	Multicenter RCT	Secukinumab vs. placebo	Analysis of blinded patient data showed a very low probability of the study meeting the primary efficacy endpoints.
Tocilizumab (TCZ) [109]	Anti-IL-6R MAB	32 patients with active, moderate-to-severe corticosteroid-resistant TED	Multicenter RCT	TCZ vs. placebo	93.3% of the patients receiving tocilizumab had a change of at least 2 in the CAS from baseline to week 16 of vs. 58.8% receiving a placebo. Tocilizumab was more effective in improving the EUGOGO-proposed composite ophthalmic outcome at week 16 (*p* = 0.03) and in reducing the size of exophthalmos from baseline to week 16 (*p* = 0.01).
Tocilizumab (TCZ) [113]	Anti-IL-6R MAB	A total of 12 trials with 448 patients were included	Meta-analysis		TCZ was most likely to be the best treatment in terms of response and proptosis reduction, followed by TMB and RTX. TMB was most likely to be the best treatment in terms of improving diplopia, followed by TCZ and RTX. TCZ had the best safety profile, followed by RTX and TMB.
Teprotumumab (TPT) [116,117]	Anti-IGF-1R MAb	Phase II: 88 patients with active moderate-to-severe TED Phase III: 83 patients with active moderate-to-severe TED	Two multicenter RCTs	TPT vs. placebo	Taking both phases into account, 73% of patients in the TPT groups (vs. 14% in the placebo groups) responded, with improvements both in CAS and exophthalmos. Improvements in proptosis of ≥2 mm occurred in 77% of patients in the TPT-treated groups and in 15% of patients in the placebo group at week 24.
Teprotumumab (TPT) [NCT04583735]	Anti-IGF-1R MAb	Phase IV: 62 patients who lived with TED from 2 to 10 years prior to the study and had low CAS	Multicenter RCT	TPT vs. placebo	TPT treatment was associated with a reduction in proptosis of 2.41 mm from baseline compared with 0.92 mm in those receiving placebo (*p* = 0.0004). In addition, 62% of teprotumumab-treated patients experienced clinically significant improvement at week 24 compared with 25% of placebo-treated patients (*p* = 0.0134).
K1-70 [114]	The TSHR antagonist	Phase I: 18 patients with GD, stable on anti-thyroid drug	Open label study	Single intramuscular or intravenous dose of K1-70	There were clinically significant improvements in symptoms of both GD (reduced tremor, improved sleep, improved mental focus, reduced toilet urgency) and TED (reduced exophthalmos measurements, reduced photosensitivity).

Anti-IL-17A Mab: anti-interleukin-17 monoclonal antibody; anti-CD20 Mab: anti-CD20- monoclonal antibody; anti-BAFF Mab: anti-BAFF monoclonal antibody; anti-IL6R Mab: anti-interleukin-6 receptor monoclonal antibody; anti-IGF-1R Mab: anti-insulin-like growth factor 1 monoclonal antibody; BAFF: B-cell activating factor; CAS: clinical activity score; ivMP: intravenous methylprednisolone; RCT: randomized controlled trial; TED: thyroid eye disease.

## 4. Summary

A better understanding of the multifactorial pathogenesis of TED contributes to the development of more effective therapies. The ideal treatment should slow progression of the disease with as little interference with patient immunity as possible. Advances in genetic engineering will enable the production of new targeted drugs. Biological therapies have shown promise, especially teprotumumab (anti-IGF-1R), which effectively reduces proptosis. Tocilizumab (anti-IL-6R) has beneficial effects on both inflammation and proptosis. The strategy of restoring tolerance to autoantigens may be the most optimal approach.

## Data Availability

Not applicable.
